# A Flow Stress Model of the AA3104-H19 Alloy for the FEM Simulation of the Beverage Can Manufacturing Process under Large Plastic Deformations

**DOI:** 10.3390/ma14216408

**Published:** 2021-10-26

**Authors:** Przemysław Wędrychowicz, Piotr Kustra, Marek Paćko, Andrij Milenin

**Affiliations:** 1 CANPACK S.A., Business Support Service, 32-800 Brzesko, Poland; przemyslaw.wedrychowicz@canpack.com; 2 Faculty of Metals Engineering and Industrial Computer Science, AGH University of Science and Technology, 30-059 Kraków, Poland; packo@agh.edu.pl (M.P.); milenin@agh.edu.pl (A.M.)

**Keywords:** flow stress, large strains, beverage can, AA3104-H19 alloy, manufacturing process

## Abstract

This paper discusses the development of a flow stress model to simulate the AA3104-H19 alloy under the conditions of large plastic deformations characteristic of the beverage can manufacturing process. This study focuses on the first five steps of this process: cupping, redrawing, ironing #1, ironing #2, ironing #3. These are the stages that reduce the thickness of the base material to the maximum, resulting in an effective strain of more than 2.0, unattainable in conventional plastometric tests. To solve this problem, the specific calculation-experimental method for the development of the flow stress model was proposed. Based on the FEM modeling of the technological process, data on the history of deformation and the trajectory of movement of the selected points of the material at all stages of the production were obtained. Microspecimens for the tensile tests were taken from the points of the beverage can wall that were determined in this way. The initial strain of each sample was taken from the FEM simulation. In this way, the tensile curves were obtained for the material points at different stages of the production. The processing of these curves allowed the creation of a flow stress model for large strains, corresponding to production conditions. The tensile tests were performed on a Zwick Z250 machine at room temperature and strain rate of 0.005 s^−1^. The FEM-based algorithm for the determination of empirical coefficients of the analytical flow stress model is presented. The final flow stress model covers the range of effective strain from 0–2. Validation of the developed model based on the measured beverage can thicknesses showed that a flow stress model was developed that correctly and accurately describes the forming process.

## 1. Introduction

The first drawn and ironed manufacturing process of a two-piece beverage can dates back to 1963. Like many metal forming processes, this one is also subjected to a numerical analysis. Numerical simulations can be helpful in the can manufacturing processes for optimization of the material hardening exponent [1], to determine the friction coefficient in the ironing process [2], force and stress level reduction during ironing [3], to investigate cup wall thickness after deep drawing [4], to reduce material consumption [5], to improve the shape of the can dome to increase its inside pressure resistance [6], or for the development of new tool designs [7]. For the simulation results to be reflected in reality, a material model must be defined correctly. This model should be defined to cover a range of parameters (temperature, strain, strain rate, etc.) relevant to the process. The work in [8] was devoted to the process of a numerical simulation for beverage can manufacturing. The authors of this paper were forced to use a different material model for the simulation because, as was mentioned, the one for AA3104-H19 was not yet available. There are many papers in which the flow curves for this alloy are presented with the effective strain limited, for example to 0.02 [9] or, in the case of paper [10], to 0.3, which is far too small from the point of view of the entire technological process. Beverage cans are typically made from 250 to 240 µm thick sheet metal and their wall thickness after the last forming stage is reduced to about 90 µm. From a simple calculation, it can be seen that the amount of the effective strain resulting from the change in the wall thickness is about 1.5. Paper [11] presented the cup modeling stage. As shown by the numerical analysis, the effective strain in the cup is about 0.6, and the presented flow curve determined experimentally covered only the range of the effective strain up to 0.1. A numerical model of the flow stress of the AA3104-H19 alloy was also presented by the authors of work [12]. Unfortunately, the paper does not provide information on the plastometric tests used to develop the parameters of this equation. This makes it impossible to determine the range of deformations for which this flow stress model was developed. An analysis of the entire beverage can forming process was made in paper [13]. However, the authors did not present a flow stress model, stating only that the material data should be entered into the DYNAFORM software.

Thus, the literature review shows that there is a lack of a flow stress model characterizing the range of the effective strain during the beverage can forming process. The presented models are based on a material tensile test followed by the extrapolation of the experimental data. These models can correctly represent the flow behavior of the material for small plastic deformations, but their error for large plastic deformations can be significant.

It is possible to develop a flow stress curve covering the presented range of the effective strain, but it requires taking and analyzing samples from each forming stage of the beverage can. Unfortunately, the size of samples that can be taken from the side of a beverage can is much smaller than that presented in the standard PN-EN 10002-1+AC1. Miniaturization of specimens for mechanical testing is reported in the technical literature. The results of study [14] show that a miniaturized specimen can be used for the mechanical property analysis in situations where tensile testing using a standard specimen is practically impossible. Farrukh et al. [15] showed that the strain–stress behavior of the miniature sample is comparable to the standard tensile test behavior. This was also confirmed by the authors of the work [16]. Additionally, three different miniaturized specimens were also partially validated by Bergonzi, Vettori, and Pirondi [17], determining that the stress levels achieved for different geometries are comparable. This means that it is possible to use miniaturized samples as long as they are validated with normative samples.

The purpose of this paper was to develop and validate of a flow stress model of the AA3104-H19 alloy for the range of the effective strain, which corresponded to the beverage can manufacturing technology.

## 2. Materials and Methods

The aluminum alloy 3104, H19 temper, due to physical and chemical properties, is usually used in the production process of pop cans. Properties of the aluminum alloy that are desirable as a material for beverage cans are low density and corrosion resistance [18]. The AA3104 alloy provides high strength to a two-piece beverage can, compatible with good forming characteristics. The main alloying elements in AA3104 are Mn, which increases its mechanical properties, and Mg, which improves its strain-hardening capacity without affecting the corrosion resistance. Additionally, to improve the mechanical properties of AA3104, the alloy is cold rolled to the H19 temper. This process strengthens the material by increasing the dislocation density and also by changing the crystallographic texture components. Other important aspects of pop can material are low earing tendency, low tendency to cause pick-up on tools, consistent thickness, width, straightness, and flatness to satisfy precise can-making tools [19]. The chemical composition analysis of the AA3104 alloy was performed using X-ray fluorescence spectrometer EDX-8100 and results are presented in Table 1.

### 2.1. Technological Process

**Figure 1:**
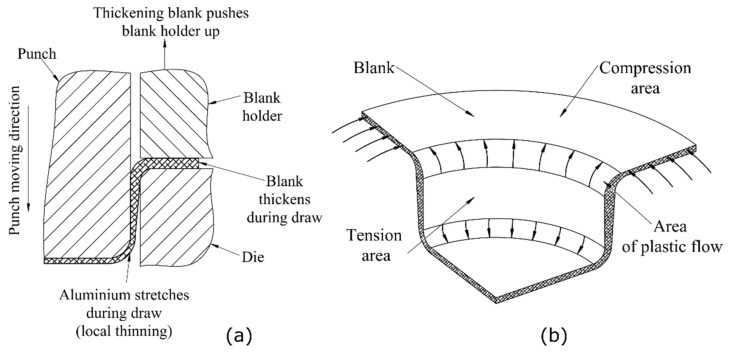
(**a**) Draw die assembly during the forming process; (**b**) the forces occurring in the material during the cup forming process.

The first step of the beverage can forming process is performed on a double-action vertical press. During each stroke of the press, two processes are completed—firstly, the blank of the raw material is cut, followed by the deep-drawing process. The cup forming process begins at the moment when the cupper punch reaches the center of the blank. To prevent the blank material buckling or wrinkling during the forming process, the material is compressed by a force applied to the blank holder draw pad. Figure 1a illustrates the cup forming process with typical drawing tools of the cupper press. Displacement of the central part of the blank caused by the punch and the force applied by the blank holder generates tension in the cup wall, which is no longer between the drawing die and the blank holder. Part of the blank, compressed by the blank holder, is thickening during the whole cup drawing process. Thickening of the blank part clamped between the draw pad and the die is induced by compression forces [21] generated in the material (Figure 1b).

Many factors have an influence on the final cup shape and its wall thickness distribution, such as the tool geometry, the punch-die clearance, the blank-holding force, the friction between the blank and the tools, and the lubrication conditions. The blank-holding force is one of the most important factors, which, on the one hand, prevents material bucking and wrinkling and, on the other, increases the friction force between the blank and the tool. This increases the drawing force and also has an impact on material thinning or in the worst case on material tearing [22].

**Figure 2:**
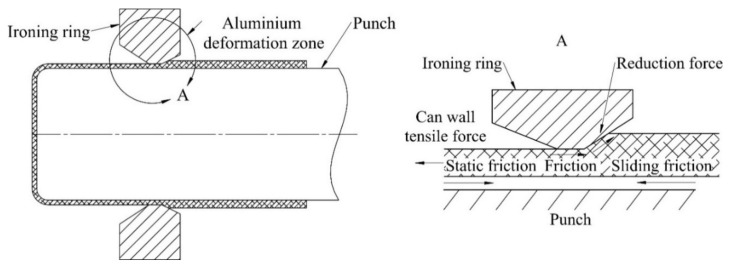
Punch and ironing die of the bodymaker machine.

**Figure 3:**
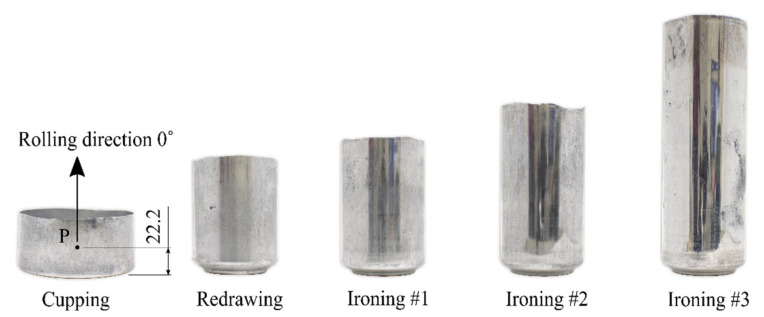
First five steps of the forming process of a beverage can.

After the cupping process, the next forming station is the bodymaker machine. Each cup formed on the cupper press is transferred via a cup conveying system to the bodymakers. The first stage of the bodymaker forming process is redrawing, in which the cup diameter is reduced to the final can diameter. After redrawing, the can moves to three wall ironing steps performed by the same bodymaker stroke as the redrawing process (Figure 2). During the ironing process, the can wall is compressed when it is pulled to the deformation area created between the bodymaker punch and the ironing ring, see Figure 2. Wall thickness reduction on each ironing die results in the corresponding can lengthening. At the end of the bodymaker stroke, the ironed can is pressed against the doming punch. Figure 3 presents the can shape after each of the first five forming steps of the beverage can production process.

The development of the flow stress model for the AA3104 alloy can be done based on the data from the tracking sensor (material point) P on the surface of the can. In addition, this point is always located on the wall of the can in a place which, according to the flow line of the material, is in the direction consistent with the direction of the rolling of the sheet. Determination of the positions and the effective strain in the tracking sensor can be performed based on the FEM simulation of the technological process. Experimental determination of the angular location of the point P on the can wall was also possible because the rolling lines are visible on the surface even after the can forming process. The location of the material point P during the can forming process will determine the position of the samples for the plastomeric tests. The initial location of the material point P on the cup was determined to be 22.2 mm from the bottom surface (Figure 3). This location was chosen to make it possible to cut a miniaturized specimen in all forming steps.

### 2.2. Existing Flow Stress Model of the AA3104-H19 Alloy for Large Plastic Deformations

**Figure 4:**
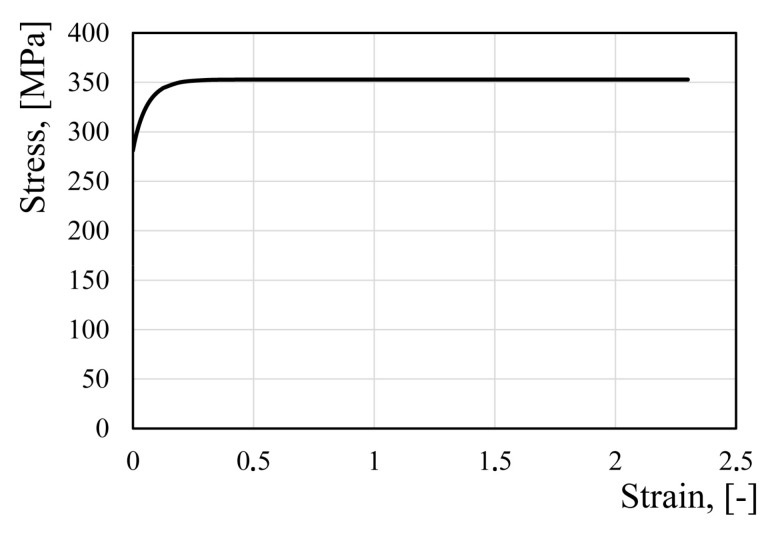
Existing Hocket–Sherby model of the flow stress of the AA3104-H19 alloy.

A preliminary numerical analysis of the technological process shown in Figure 3 was performed in the LS Dyna software using the existing flow stress Hocket–Sherby [23] model with parameters delivered by one of the AA 3104 H19 sheet manufacturers:

(1) $$\sigma =B-\left(B-A\right)exp\;\left(-C\varepsilon^{n}\right),$$

where *ε* is effective strain, *A* = 281.25, *B* = 351.9, *C* = 14.99, and *n* = 0.9691. The flow stress model is shown in Figure 4. As concluded from Figure 4, this model does not predict material hardening during the forming process under large plastic deformations.

### 2.3. Numerical Simulations

**Figure 5:**
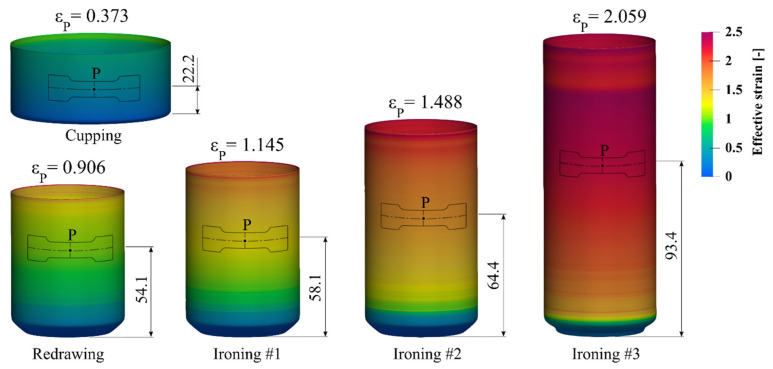
Distribution of the effective strain in the first five steps of the beverage can forming process with an existing model of flow stress.

**Figure 6:**
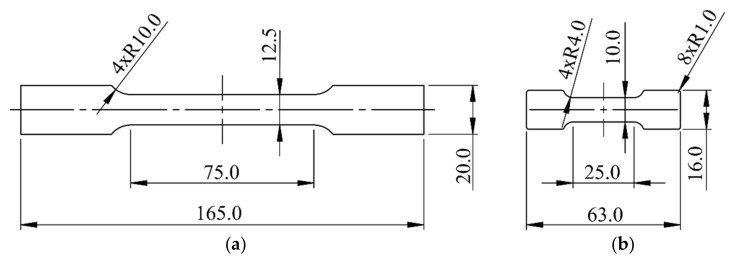
Samples for the tensile tests: (**a**) standard sample (PN-EN 10002-1+AC1); (**b**) modified (minimized) sample.

The FEM simulation of the beverage can forming process was performed using a 2D axisymmetric model, which was prepared in the LS-Prepost preprocessor, dedicated for the LS-Dyna solver that was used. Results visualizations were prepared in an open-source, multi-platform ParaView application. Preliminary simulations were focused on determining the optimum number of finite elements on the can wall. As was observed, an increasing number of finite elements of wall thickness results in a lower final wall thickness; this is caused by, among other factors, a small forming zone on the ironing dies. After a series of simulations with different numbers of finite elements on the wall thickness, it was determined that once there were more than nine elements of wall thickness of the can, its final thickness after the simulation stopped changing. Therefore, it was decided to use models with 10 elements of can wall thickness. The contact card *2D_AUTOMATIC_SURFACE_TO_SURFACE was used for all contact pair definitions [24]. The forming tools were modeled as rigid bodies with discrete shell elements represented (4 integration points, type = 15) [24]. The static and dynamic friction coefficients between the sheet and the tools were assumed to be *μ_S_* = 0.1 and *μ_D_* = 0.05, respectively. For contact between the sheet and the punch, static and dynamic friction coefficients were accordingly *μ_S_* = 0.15 and *μ_D_* = 0.1 [25]. The AA3104 blank material with an initial thickness of 0.240 mm was used. The flow stress of this material in LS Dyna was defined by the Hocket–Sherby model (Equation (1)). The blank holder force for the cup drawing step was set to 9.3 kN and in the next redrawing step increased to 24 kN. Based on those parameters, the preliminary FE simulations of the first five stages of the beverage can forming process were performed. Material point P on the surface of the can was traced during the calculations, see Figure 5. The localization of point P on the wall of the can at different stages of the forming process determined the location of sampling for the plastometric tests. The value of the effective strain at this point is presented in Figure 6. The samples for the plastometric tests were cut from the blanks at all stages of the forming process using a die cutter, which prevented the occurrence of residual stress.

### 2.4. Development of Specimens for the Plastometric Tests

For the studied material with a thickness of 0.100–0.250 mm, only tensile tests were considered as the plastometric tests. The problem as it appeared at the beginning of this analysis was related to the size of the standard samples used for the tests. The standard sample for tensile tests is long enough to cover material zones that are aligned and perpendicular to the rolling direction. Since this analysis was to be carried out on local samples, it was decided to miniaturize the sample. Figure 6 shows the standard sample and the proposed miniaturized sample.

### 2.5. Calibration of the Flow Stress Model

**Figure 7:**
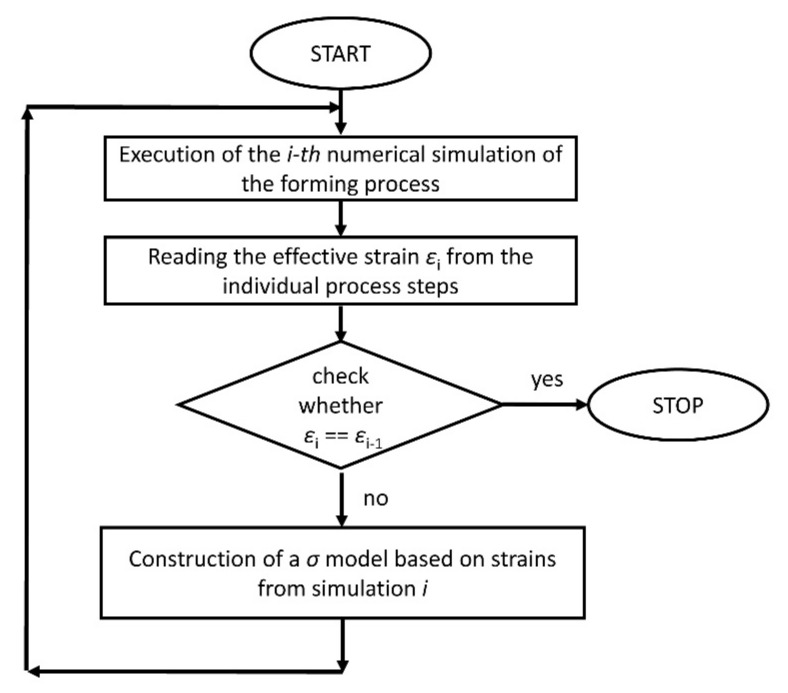
Scheme of the fitting algorithm for determining the parameters of the flow stress model.

Due to the large effective strain in the analysis process, it was not possible to use the classical inverse method as it was presented in work [26]. To develop a flow stress model of the AA3104-H19 aluminum alloy, the following algorithm was proposed. The preliminary numerical analysis, presented in Figure 5, allowed for tracking and determining the location of material point P at each stage of the forming process. Samples for the plastometric tests were cut from the material based on the location of point P. Based on the current plastometric data and the effective strain at point P, data for the new flow stress model were obtained. Using a current flow stress model, numerical simulations of the technological process were performed and the new strain values at material point P were determined. The block diagram of the fitting algorithm is shown in Figure 7.

## 3. Results of the Experimental Study

The plastometric tensile tests were performed on a Zwick Z250 testing machine at room temperature using a strain rate of 0.005 s^−1^. The specimens were stretched to failure.

### 3.1. Tensile Tests of the Blank Material

**Figure 8:**
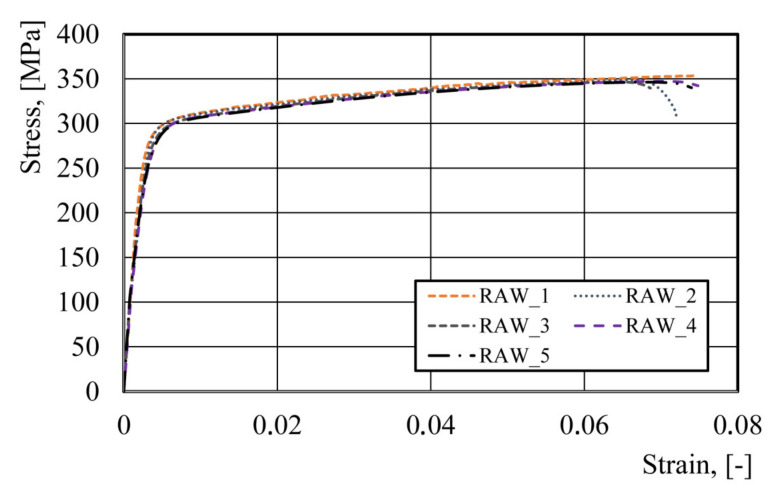
Stress–strain curves for the miniaturized samples of the raw material.

In the first stage of the study, comparative tests were performed on the standard specimen and the miniaturized specimen. Ten tensile tests were performed on the blank (raw material). The results of the analysis are shown in Table 2. The analysis of the tensile tests shows that the flow stress–strain curves practically coincided; the standard deviation was 0.64 MPa and 3 MPa for the yield stress (YS) and the ultimate tensile strength (UTS), respectively. For measurement of the longitudinal strain, mechanical extensometers were used, which eliminated the sample scale effect. The results presented in Table 2 suggest that the miniaturized sample correctly represents the ductile and strength characteristics of the material. Therefore, the further part of the research was done on the miniaturized samples. Figure 8 shows the plastometric data for the raw material.

The Hansel–Spittel [27] equation was used to develop a new flow stress model of the AA3104-H19 alloy. The choice of the Hansel–Spittel equation to create a new flow stress model was due to two circumstances. Firstly, this equation is widely used in the practice of modeling metal forming processes. For this reason, the developed model can be used in various FEM programs, for example, in QForm or Forge3. Secondly, the Hansel–Spittel equation contains a sufficiently large number of coefficients to potentially approximate more complex flow stress–strain dependences than that shown in the Figure 4 curve.

For the isothermal conditions (cooling during the process with a heat carrier to a temperature of about 40–45 °C) and a lack of sensitivity to strain rate, this equation was simplified to the following form:

(2) $$\sigma =A\varepsilon^{m_{1}}\exp\left(\frac{m_{2}}{\varepsilon }\right){\left(1+\varepsilon \right)}^{m_{3}}exp\left(m_{4}\varepsilon \right),$$

where *A*, *m*_1_–*m*_4_—empirical parameters.

Testing of samples from different stages of the beverage can manufacturing process was performed using a minimum of two replicates for each sample. If the difference in the YS or UTS values was greater than 2%, a third test was performed. The means and standard deviations of the tests are provided in Table 3.

**Figure 9:**
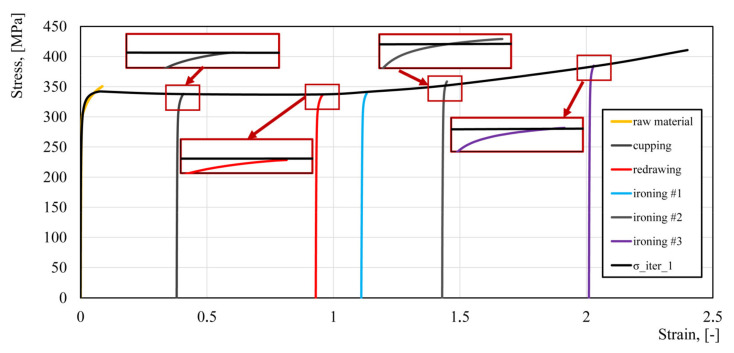
Approximation of the plastometric data for the first iteration of the fitting algorithm.

The flow stress–strain curves for each stage of the beverage can forming process were shifted according to the deformations obtained from the numerical simulation (Figure 5). The parameters of the new flow stress model (2) were determined using an approximation of the experimental data (Figure 8 and Table 3) with minimization of the goal function δ:

(3) $$\delta =\overset{n}{\underset{j=1}{\sum }}\overset{m}{\underset{i=1}{\sum }}{\left(\sigma_{ij}^{calc}-\sigma_{ij}^{exper}\right)}^{2}\to min$$

where $\sigma_{ij}^{calc}$—stress calculated by Equation (2), $\sigma_{ij}^{exper}$—the stress value measured on the testing machine, *m*—number of points in a single tensile test, and *n*—number of the tensile tests. The whole yield stress curve for the blank was taken for approximation, while only the UTS and the corresponding strains for other technological stages were used. Empirical parameters of the flow stress model are presented in Table 4 and the obtained flow stress–strain model is shown in Figure 9. Based on Equation (3), empirical parameters of Equation (2) were determined and are presented in Table 4.

### 3.2. Calibration of the New Flow Stress Model

**Figure 10:**
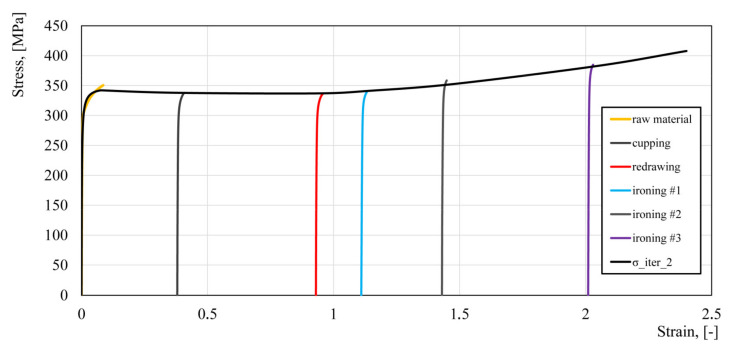
Approximation of the plastometric data—second iteration.

Based on the developed flow stress model, numerical calculations of the beverage can production process were performed again. The effective strain values read from the numerical simulation at point P were: cupping—0.384, redrawing—0.939, ironing #1—1.139, ironing #2—1.482, ironing #3—2.052. As can be seen from the data presented in Figure 5, the effective strain values for the last two stages of the beverage can forming process are significantly different (ironing #2—1.43, ironing #3—2.01). Therefore, it was necessary to perform the second iteration of the fitting algorithm. Based on the effective plastic strain, the flow curves from each forming step were shifted according to the strain value and then the process of approximating the experimental data was performed again. The results of the fitting algorithm are presented in Table 5 and Figure 10.

**Figure 11:**
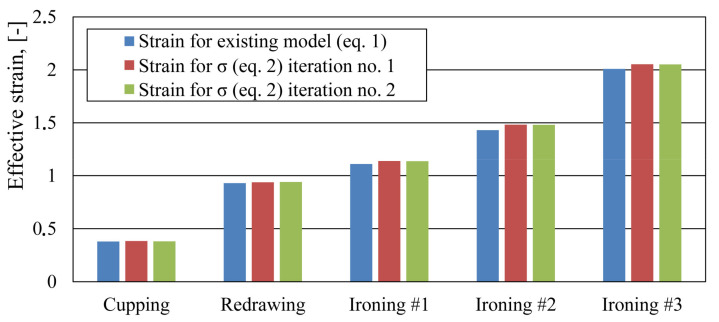
Effective strain values from the numerical simulation for material models (1) and (2) from the first and second iteration of the fitting algorithm.

**Figure 12:**
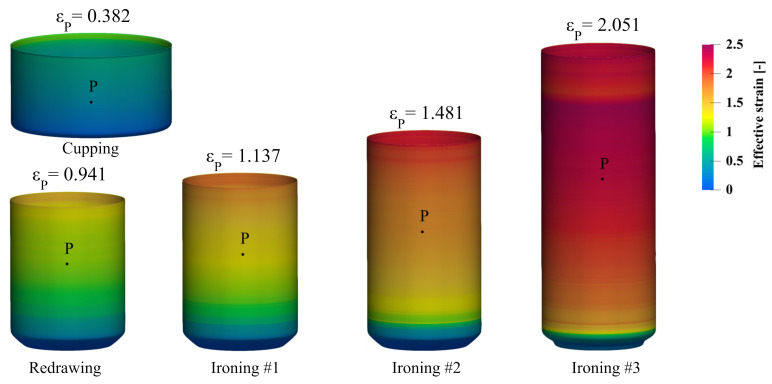
Distribution of the effective strain in the first five steps of the beverage can forming process with developed model (2) and parameters from Table 5.

Numerical simulation of the beverage can forming process was performed using material model (2) with parameters from Table 5. The effective strain values for all stages of the fitting algorithm are shown in Figure 11. As can be seen from those results, the strain values for the first and second iteration of the fitting algorithm were practically identical. This means that the fitting algorithm was rapidly converging. The strain distribution in the beverage can body at all stages of the forming process are shown in Figure 12.

**Figure 13:**
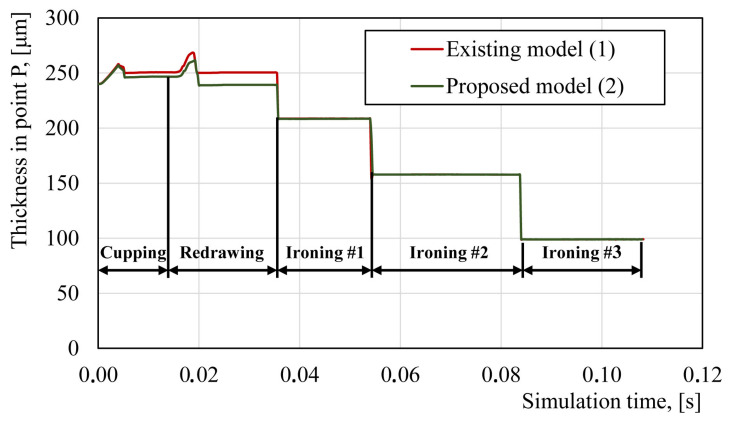
Comparison of the thickness value at point P for the existing flow stress model (1) and the developed model (2).

The validation of the new flow stress model was performed based on the can wall thickness at point P in the analyzed stages of the beverage can forming process. As can be seen from the data presented in Figure 13 and Table 6, the developed model more accurately described the thicknesses in the material for the first five stages of the beverage can forming process, during which, reduction of the base material thickness was the highest.

## 4. Discussion

As was described earlier, the forming process of the can body requires several forming steps. This multistage forming process leads to the accumulation of large deformations in the AA3104-H19 material. Additionally, during the forming process, the material is subjected to different loading conditions. Figure 1a,b illustrates two areas of the formed can with tensional and compressional stress states. The compression stress state generated under the blank holder results in material thickening, and when the material is released from the blank holder tool, it moves to the area between the punch and the draw die where the tensional forces stretch the material, causing its thinning. Cyclic loading of point P, which can also be observed as thickness change during the cupping and redrawing steps (Figure 13) is a reason for material softening, also called the Bauschinger effect. The occurrence of this phenomenon was investigated for the 3104 aluminum alloy in the H19 condition by J.,-L. Duval et al. in [28]. Other authors [29] studied the Bauschinger effect in the incremental forming of the 5754 aluminum alloy and showed its influences on the multistage forming process. For further beverage can body shape optimization and technological process improvement by FEM, input data, such as those from the flow stress model, must be provided as accurately as possible for the best representation of the material’s behavior. The literature also lacks the flow stress curves of the AA3104 alloy for large plastic deformations, such as those observed in the production process of a beverage can. There are papers in the literature using numerical models for this purpose for the maximum effective strain of 0.02 [9], 0.1 [11], or 0.3 [10]. These curves are then extrapolated for large deformations in the FEM software. Such models may be subject to large numerical errors and they may not take into consideration the phenomena occurring in the material. The developed flow stress model takes into consideration the Bauschinger effect in deep drawing operations of the can forming process and also characterizes the described material flow in the whole range of strain levels in the beverage can forming process. The developed flow stress model could be suitable for use in can body simulation manufacturing according to the presented technology. Although only one specific can size was used in the conducted research, there are currently many sizes of cans produced from aluminum alloy 3104 H19 sheets of 240 micrometers thick. The variety of can sizes and increasing precision of forming machines and tools provides a range of opportunities to use the developed curve in simulation models of new can body shapes. It is also possible to use this model for numerical simulations of necking and reforming processes. Validation of the developed model based on the can wall thicknesses at different stages of the forming process showed that the model was correctly developed. Therefore, this model can be used in the optimization of can forming process or in the can wall thinning process.

## 5. Conclusions

1. A methodology was developed to determine the flow stress model of the AA3104-H19 alloy for large plastic deformations;
2. Since it was not possible to cut the standard samples for the plastometric tests from the wall of the beverage can, a miniaturized specimen was developed and verified;
3. A numerical model of the beverage can manufacturing process was used to determine the locations of the samples for the plastometric tests and the effective strain in the center of those samples;
4. The developed flow stress model covers the range of the actual effective strain in the beverage can manufacturing process up to 2.0. This model also takes into consideration the deformation history and the Bauschinger effect;
5. The developed model describes the flow characteristics of the material much more effectively than the model provided by the sheet metal supplier. This results in a better representation of the material thickness in the simulation process. On the cup and redraw operations, the difference in thickness relative to the actual thickness decreased from 5 to 1 and 11.5 to 0.5 µm, respectively;
6. The presented flow stress model can be used in the development process of beverage can shaping and manufacturing technology.

## Figures and Tables

**Table 1:** Chemical composition (in wt%) of the AA3104 alloy [20].

Grade Designation	Si	Fe	Cu	Mn	Mg	Zn	Ga	V	Ti	Unspecified Other Elements		Al
										Each	Total	
3104	0.176 ± 0.009	0.431 ± 0.003	0.129 ± 0.001	0.833 ± 0.005	1.191 ± 0.020	0.032 ± 0.001	0.009 ± 0.001	0.031 ± 0.002	0.015 ± 0.002	-	0.071 ± 0.002	97.082 ± 0.162

**Table 2:** Summary of the plastometric tests for the standard and miniaturized samples for the raw material.

Sample Type	*a*_0_ (mm)	*b*_0_ (mm)	*S*_0_ (mm^2^)	YS (MPa)	UTS (MPa)	A (%)
Standard sample	0.240 ± 0.001	12.51 ± 0.03	3.008 ± 0.005	286.74 ± 1.43	315.31 ± 1.16	4.92 ± 0.36
Miniature sample	0.240 ± 0.001	10.00 ± 0.07	2.404 ± 0.019	286.10 ± 0.93	318.33 ± 0.33	6.10 ± 0.39

*a*_0_—sample thickness, *b*_0_—sample width, *S*_0_—a cross-section of the sample.

**Table 3:** Average values and standard deviations of material characteristics measured at different stages of the beverage can forming process.

Forming Step	*a*_0_ (mm)	*b*_0_ (mm)	YS (MPa)	UTS (MPa)	A (%)
cupping	0.245 ± 0.0010	10.01 ± 0.01	293.65 ± 1.05	328.10 ± 0.60	2.46 ± 0.06
redrawing	0.239 ± 0.0015	10.01 ± 0.01	290.15 ± 0.85	325.95 ± 0.85	1.52 ± 0.48
ironing #1	0.206 ± 0.0005	9.98 ± 0.03	301.20 ± 5.92	330.97 ± 0.33	1.41 ± 0.33
ironing #2	0.158 ± 0.0005	9.92 ± 0.01	320.70 ± 0.20	351.00 ± 0.60	0.92 ± 0.08
ironing #3	0.099 ± 0.0005	9.86 ± 0.05	345.80 ± 2.60	374.05 ± 2.65	0.72 ± 0.12

**Table 4:** Parameters of the (Equation (2)) model for the first iteration of the fitting algorithm.

*A*	*m_1_*	*m_2_*	*m_3_*	*m_4_*
377.524	0.024674	−0.00088	−0.73833	0.4027

**Table 5:** Parameters of the (Equation (2)) model for the second iteration of the fitting algorithm.

*A*	*m_1_*	*m_2_*	*m_3_*	*m_4_*
376.974	0.02430	−0.000879	−0.7217	0.3921

**Table 6:** Comparison of the thickness of the beverage can wall at material point P for the numerical models and measurement.

Numerical Models and Measurement	The Thickness of Can Wall in Material Point P (µm)				
	Cupping	Redrawing	Ironing #1	Ironing #2	Ironing #3
Existing flow stress model (Equation (1))	250	251	209	158	99
Proposed flow stress model (Equation (2))	246	239	208	158	99
Mean value of measured specimens	245	239.5	205.7	157.5	98.5

## Data Availability

Data are provided within the article.
